# Supplementary material for: Development of a computer-aided design software for the quantitative evaluation of aesthetic damage
Source: PLoS One. 2019 Dec 18;14(12):e0226322. doi: 10.1371/journal.pone.0226322 (PMC6919621; doi:10.1371/journal.pone.0226322)
Supplement: S1 Code site — 1. (ZIP) [file pone.0226322.s001.zip › [EESC_jr][Projeto]Relatorio_07_21/vendors/select2/docs/_includes/options/dropdown/tagging.html]

## Can options be created based on the search term?

### How do I enable tagging?

{% highlight js linenos %}
$('select').select2({
tags: true
});
{% endhighlight %}
{% include options/not-written.html %}

### Does tagging work with a single select?

Yes.

{% include options/not-written.html %}

### How do I add extra properties to the tag?

{% highlight js linenos %}
$('select').select2({
createTag: function (params) {
var term = $.trim(params.term);
if (term === '') {
return null;
}
return {
id: term,
text: term,
newTag: true // add additional parameters
}
}
});
{% endhighlight %}
{% include options/not-written.html %}

### Can I control when tags are created?

{% highlight js linenos %}
$('select').select2({
createTag: function (params) {
// Don't offset to create a tag if there is no @ symbol
if (params.term.indexOf('@') === -1) {
// Return null to disable tag creation
return null;
}
return {
id: params.term,
text: params.term
}
}
});
{% endhighlight %}
{% include options/not-written.html %}

### How do I control the placement of the option?

{% highlight js linenos %}
$('select').select2({
insertTag: function (data, tag) {
// Insert the tag at the end of the results
data.push(tag);
}
});
{% endhighlight %}
{% include options/not-written.html %}
